# Myeloid-derived suppressor cells in colorectal cancer: prognostic biomarkers and therapeutic targets

**DOI:** 10.37349/etat.2022.00097

**Published:** 2022-08-31

**Authors:** Mohammad A. Al-Mterin, Eyad Elkord

**Affiliations:** 1 Natural and Medical Sciences Research Center, University of Nizwa, Nizwa 616, Oman; 2 Biomedical Research Center, School of Science, Engineering and Environment, University of Salford, M5 4WT Manchester, UK; Université Paris-Saclay, France

**Keywords:** Myeloid-derived suppressor cells, colorectal cancer, prognostic biomarkers, targeting

## Abstract

Myeloid-derived suppressor cells (MDSCs) are a group of immature myeloid cells, which are expanded in most cancer patients. MDSCs suppress host immune responses, leading to cancer growth and progression. Several studies demonstrated that there was a relationship between levels of MDSCs and tumorigenesis in colorectal cancer (CRC) patients. MDSCs are now being investigated for their role as possible therapeutic targets in cancer treatment. This review summarizes available studies that investigated MDSC expansion in CRC patients, as well as their role in CRC tumorigenesis, prognosis, and targeting. Based on the available studies, there is a possible relationship between high levels of MDSCs and CRC progression. Additionally, targeting MDSCs in CRC patients selectively represents a significant challenge for the development of targeted treatments. Targeting of MDSCs could be exploited in different ways including MDSC depletion, inhibition of MDSC function and recruitment, and enhancing MDSC differentiation. Overall, MDSCs could be exploited as prognostic biomarkers and potential therapeutic targets in CRC.

## Introduction

Myeloid-derived suppressor cells (MDSCs) are a heterogeneous group of immature myeloid cells, which are characterized by strong abilities to suppress both innate and adaptive immune responses, especially T cell functions [1, 2]. MDSCs are critical immune regulatory cells, expanding in response to various pathological conditions such as cancer, inflammation, and infection [2].

In healthy individuals, immature myeloid cells differentiate into granulocytes, macrophages, or dendritic cells (DCs). However, during acute infections, immature myeloid cells increase and differentiate mainly into activated neutrophils and monocytes [3]. This process is critical to protect the host cells from pathological illnesses. During cancers and chronic inflammations, immature myeloid cells are characterized by a continual release of inhibitory signals [4]. Consequently, the accumulation of immature myeloid cells leads to incomplete differentiation of these cells into activated neutrophils and monocytes [2]. Over time, the long-term inflammatory signals create the environment for increasing and activation of MDSCs [5, 6].

MDSCs in human are defined as CD33^+^CD11b^+^HLA-DR^low^ cells, and according to cell surface markers and cell morphology, human MDSCs may be divided into two main subgroups: granulocytic/polymorphonuclear MDSCs (G-MDSCs/PMN-MDSCs, CD33^+^CD11b^+^HLA-DR^low^CD15^+^ cells), and monocytic MDSCs (M-MDSCs, CD33^+^CD11b^+^HLA-DR^low^CD14^+^ cells) [3, 7–9]. Lately, an additional subgroup has been identified as CD33^+^CD11b^+^HLA-DR^low^CD14^–^CD15^–^, and they are named immature MDSCs (I-MDSCs) or early-stage MDSCs (e-MDSCs) [3, 10, 11].

## MDSCs in cancer

MDSCs are expanded in the peripheral blood of individuals with different malignant and non-malignant diseases [12]. MDSC subgroups of granulocytic and monocytic cells have been recognized and characterized in several human malignancies [13–16], including breast cancer [17], colorectal cancer (CRC) [18–20], melanoma [21], non-small cell lung cancer [22], head and neck cancer [23], pancreatic, esophageal and gastric cancer [24], and renal cell carcinoma [25]. MDSCs inhibit anti-tumor immune responses [16, 26, 27], and as a result, cancer cells maintain their rate of progression [11]. Moreover, MDSC levels in cancer patients are considered to have prognostic and predictive values [28].

Many secreted factors such as tumor-derived granulocyte colony-stimulating factor (G-CSF), granulocyte-macrophage colony-stimulating factor (GM-CSF), macrophage colony-stimulating factor (M-CSF), stem cell factor, vascular endothelial growth factor (VEGF), and interleukin-3 (IL-3) are released in the tumor microenvironment (TME). These factors stimulate the production or expansion of MDSCs [29]. Accordingly, MDSCs have been related to the induction of T-cell dysfunction in cancer via the production of arginase (ARG) I, reactive oxygen species (ROS), peroxynitrites (ONOO^–^), transforming growth factor-beta (TGF-β), and IL-10 [1, 2, 30–32]. Additionally, MDSCs mediate the development of tumor-induced T regulatory cells (Tregs) and T helper 17 (Th17) cells [2, 33, 34].

## MDSCs in CRC

CRC is the second-deadliest cancer and the third most frequent cancer worldwide [35]. A total of 1.8 million new cases of colon cancer were diagnosed in 2018, accounting for approximately 10% of all new cancer cases and deaths globally [35, 36]. Chronic mucosal inflammation has been linked to the development of CRC [20, 37]. This association is mediated by proinflammatory cells and mediators such as prostaglandin E2 (PGE2), IL-6, VEGF, IL-1β, S100A8/A9 proteins, and the complement component 5a (C5a), which disrupt the outcome of anticancer therapy [38, 39]. Moreover, these proinflammatory mediators enhance the induction of MDSCs, which in turn increase their accumulation [40]. Over time, such accumulation leads to the suppression of immune responses and antitumor immunity, thereby enabling tumor development and progression [40].

Several studies demonstrated a relationship between levels of MDSCs and tumorigenesis in CRC patients [18–20, 41, 42]. OuYang et al. [43], reported that MDSC levels in CRC patients were elevated in peripheral blood and tumor tissues. In addition, tumor-induced MDSCs have the ability to inhibit the proliferation of T cells and promote CRC cell growth through cell-to-cell communication [43]. Also, they found a correlation between higher levels of MDSCs in peripheral blood with advanced tumor- node-metastasis (TNM) stages and lymph node metastases [43]. Moreover, Yan et al. [44], demonstrated that accumulation of γδT17 cells correlated with human CRC progression through increasing the expansion and recruitment of MDSCs. Also, they found that levels of PMN-MDSCs are abundant in tumor tissues in CRC patients [44].

**Figure 1.**
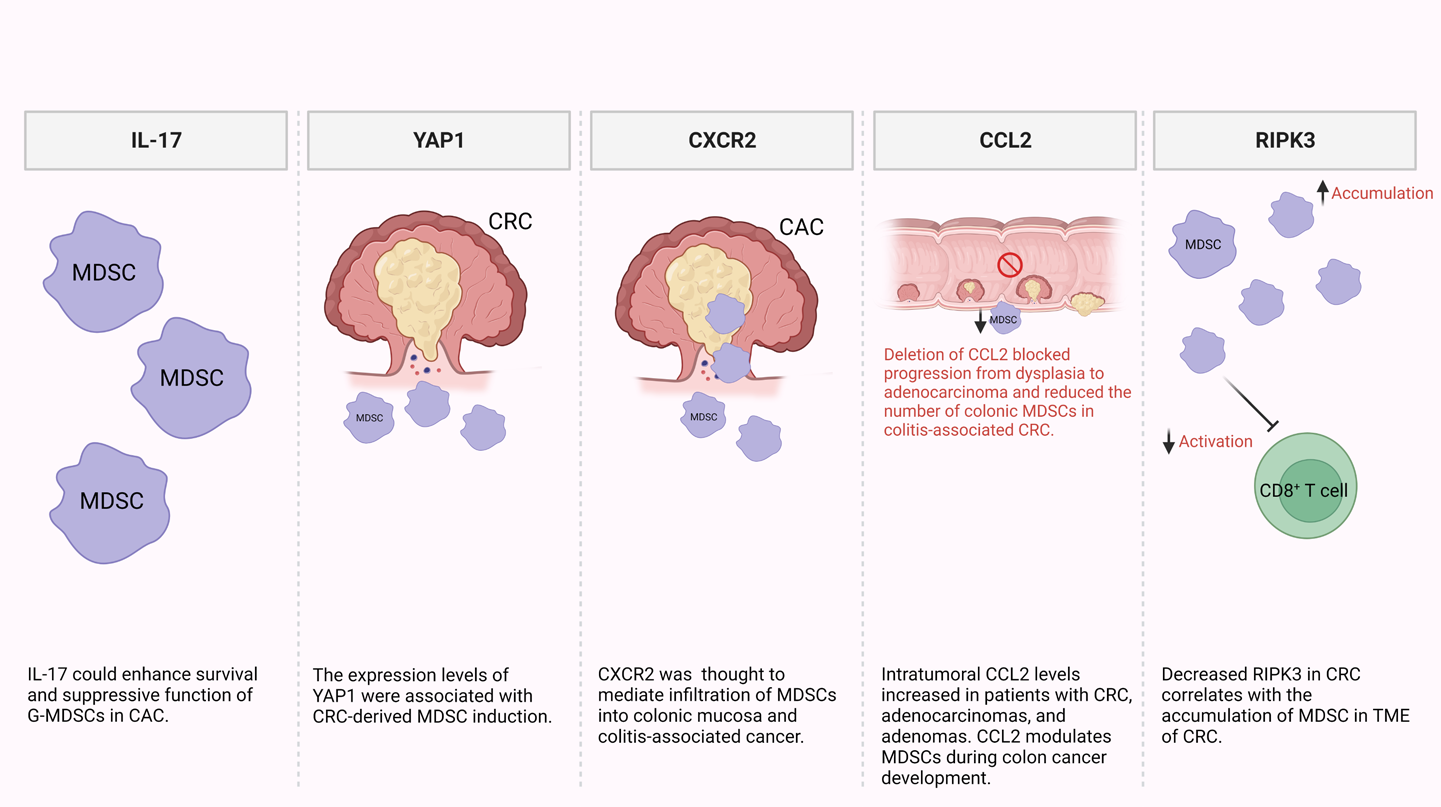
Potential mechanisms of MDSC induction and accumulation in CRC and CAC. In the TME, some factors were found to be responsible for MDSC recruitment and expansion including IL-17, YAP1, CXCR2, CCL2, and RIPK3

It has been reported that MDSCs are recruited and expanded mainly by IL-17 secreted by innate γδT (γδT17) cells (Figure 1) [44, 45]. Additionally, another study reported that IL-17 could enhance the survival and suppressive function of G-MDSCs *in vitro* in the early stage of colitis-associated cancer (CAC) [46]. Furthermore, levels of MDSCs and Yes-associated protein 1 (YAP1) were shown to be positively correlated with poor clinical outcomes, reduced disease-free survival (DFS), and overall survival (OS) in CRC patients [47]. They also reported that expression levels of YAP1 and phosphatase and tensin homologue (PTEN) were associated with MDSCs induction in CRC tumor tissues *in vitro* (Figure 1) [47]. It has been previously shown that CRC patients have significantly higher levels of granulocytic myeloid cells (GMCs) in the circulation and tumor tissues [48]. Interestingly, there was a correlation between the elevation of peripheral GMCs and a higher tumor stage [48]. Moreover, another study observed an increase in e-MDSCs (CD33^+^CD11b^+^HLA-DR^–^CD14^–^CD15^–^) in tumor tissues of CRC patients [48]. Notably, PGE2 has a role in CRC progression [49]. It promotes tumor progression by stimulation of C-X-C motif chemokine receptor 2 (CXCR2) ligand expression, which results in the induction of tumor-associated angiogenesis (Figure 1) [50]. Accordingly, in a mouse model of CAC, Katoh et al. [51], found that deletion of *Cxcr2* significantly reduced colonic chronic inflammation and colitis-associated carcinogenesis by limiting the infiltration of G-MDSCs into the colonic mucosa and tumors. Moreover, Chun et al. [52], found that C-C motif chemokine ligand 2 (CCL2) increased colorectal carcinogenesis by enhancing intratumoral PMN-MDSC accumulation. Importantly, it was observed that CCL2 promotes programmed death ligand 1 (PD-L1) expression on PMN-MDSCs, suggesting that CCL2 neutralizing agents may be useful to increase the responsiveness to current immune checkpoint therapies in some patients (Figure 1) [52]. Fědorová et al. [53], found that numbers of circulating M-MDSC were not associated with metastatic disease in advanced CRC patients. However, the numbers of circulating CD33^hi^ PMN-MDSCs were increased in patients with distant metastases [53]. Moreover, low levels of receptor-interacting protein kinase 3 (RIPK3), which is required for mucosal repair in inflammatory bowel diseases (IBDs), correlated with the accumulation of MDSCs in murine CRC [54]. Specifically, they found that PGE2 increased the immune-suppressive action of MDSCs, while also accelerating tumor development [54]. Moreover, PGE2 inhibited RIPK3 expression while increasing the expression of nuclear factor kappa B (NFκB) and cyclooxygenase-2 (COX-2) in MDSCs, suggesting that RIPK3 and PGE2 could be therapeutic targets in CRC patients (Figure 1) [54].

Besides that, Karakasheva et al. [55], found a significant increase in circulating CD38^+^ M-MDSCs, and a trend of expansion of circulating CD38^+^ PMN-MDSCs was detected in CRC patients, compared with healthy controls. Indeed, they demonstrated that circulating CD38^+^ M-MDSCs were functionally immunosuppressive and might potentially contribute to the development of CRC [55]. Interestingly, Wang et al. [56], found that G-MDSCs enhance tumor cell stemness and progression through exosomes in mice with CRC. Additionally, they found that S100A9 is over-expressed in G-MDSC-derived exosomes, and blocking it reduces CRC cell stemness [56]. Also, they reported that hypoxia stimulates G-MDSCs to produce more exosomes in a hypoxia-inducible factor 1α (HIF-1α)-dependent manner, and respiratory hyperoxia can decrease tumor cell stemness by inhibiting the production of G-MDSCs exosomes in CRC [56].

IL-8 plays an important role in gastrointestinal carcinogenesis by mobilizing immature CD11b^+^Gr-1^+^ myeloid cells [57]. IL-8 was strongly up-regulated in colorectal tumors from patients compared with normal tissues [57]. Increased levels of CD14^+^HLA-DR^–/low^ MDSCs in CRC liver metastasis patients inhibited T cell proliferation and indicated poor clinical outcomes [58]. MDSCs induced by sphingosine-1-phosphate receptor 1 (S1PR1)-signal transducer and activator of transcription-3 (STAT3) in CRC cells can promote growth and liver metastasis in CRC [58]. Obviously, levels of PMN-MDSCs and e-MDSCs were elevated in tumor tissues compared with normal tissues in CRC patients [59]. Limagne et al. [60], found that levels of Tregs, Th17, and G-MDSC were significantly elevated in metastatic CRC. Importantly, only a high level of G-MDSC was associated with a poor disease prognosis, according to this study [60]. In pre-treatment patients with metastatic CRC, high M-MDSC, low CD4^+^ T effector memory cell (T_EM_), or low CD8^+^ T_EM_ levels had significantly shorter progression-free survival [61]. The studies which demonstrated a relationship between levels of MDSCs and tumorigenesis in CRC patients are summarized in Table 1.

**Table 1.** Summary of studies, which demonstrated relationships between levels of MDSCs and tumorigenesis in CRC patients

**Sample type**	**MDSC phenotype**	**Number of samples**	**Main findings**	**References**
Peripheral blood	Lin^–/low^HLA-DR^–^ CD11b^+^CD33^+^ MDSCs	64 patients	Increased percentage and the absolute number of Lin^–/low^HLA-DR^–^CD11b^+^CD33^+^ MDSCs compared with healthy controls. This increase is closely correlated with clinical cancer stage and tumor metastasis but not primary tumor size	[20]
Peripheral blood	CD11b^+^CD33^+^HLA-DR^–^ MDSCs	23 patients with stage IV metastatic CRC	Patients with advanced CRC display enhanced MDSC levels and reduced CD247 expression	[37]
Peripheral blood and tumor tissues	CD33^+^HLA-DR^–^ MDSC	49 CRC patients	A considerable increase in the percentage of CD33^+^HLA-DR^–^ MDSCs was observed in the peripheral blood and tumor tissues of CRC patients as compared with healthy controls	[18]
Peripheral blood and tumor tissues	CD33^+^CD11b^+^HLA-DR^–^	32 age-matched healthy donors and 42 patients with CRC at the time of first diagnosis	CRC patients had elevated levels of CD33^+^CD11b^+^HLA-DR^–^ MDSCs in primary tumor tissues and in peripheral blood. These elevated circulating MDSCs were correlated with advanced TNM stages and lymph node metastases	[42]
Tumor tissues	CD45^+^Lin^–^HLA-DR^–^ CD11b^+^CD33^+^CD66b^+^	154 patients with colorectal adenocarcinoma	Activated inflammatory-DCs induced γδT17 cells to secrete IL-8, tumor necrosis factor alpha, and GM-CSF with a concomitant accumulation of immunosuppressive PMN-MDSCs in the tumor	[44]
Tumor tissues	CD33^+^CD11b^+^HLA-DR^–^ MDSCs	145 newly diagnosed CRC patients who did not accept any preoperative chemoradiotherapy	A significant association between CD33^+^ MDSC number and YAP1 and PTEN levels in CRC patients. The CD33^+^ MDSCs, YAP1, and PTEN were identified as predictors for the prognosis of CRC patients	[46]
Peripheral blood and tumor tissues	CD33^+^CD11b^+^HLA-DR^–/low^CD15^+^CD33^+^CD11b^+^HLA- DR^–^CD14^–^CD15^−^	21 CRC and 21 healthy donors	The expansion of peripheral GMCs correlated with higher stage and histological grade of cancer, thereby suggesting their role in cancer progression	[47]
Peripheral blood	M-MDSCs were detected as CD45^+^CD11b^+^CD33^+^HLA- DR^low^CD14^+^CD15^–^, G-MDSCs (CD33^hi^ PMN- MDSC) were detected as CD45^+^CD11b^+^CD33^hi^HLA- DR^low^CD14^–^CD15^+^	10 patients with advanced colorectal carcinoma	Levels of circulating M-MDSCs were not associated with metastatic disease within advanced CRC patients. Levels of circulating CD33^hi^ PMN-MDSCs were elevated in patients with distant metastases compared to T3 M0 subgroup	[52]
PBMC	M-MDSCs (defined as CD14^+^HLA-DR^–/low^) PMN-MDSCs (defined as low density, CD33^+^CD11b^+^CD14^–^ CD15^+^SSC^hi^)	1 CRC patient and 8 healthy donors	A significant expansion of CD38^+^ M-MDSCs and a trend of expansion of CD38^+^ PMN-MDSCs (accompanied by a trend of increased CD38 expression on both M-MDSCs and PMN-MDSCs) were observed in PBMCs of CRC patients when compared with healthy donors	[54]

PBMC: peripheral blood mononuclear cell

## Prognostic significance of MDSCs in CRC

MDSCs were found to be an independent prognostic factor in several types of cancer. Specifically, levels of MDSCs were shown to be associated with clinical outcomes in patients with advanced melanoma, indicating that they could have prognostic value and may be used to identify patients who are at high risk of disease progression [62]. Moreover, elevated numbers of MDSCs were shown to be correlated with more advanced cancer stages, and with decreased survival rates in gastric cancer patients [28]. Similarly, high numbers of circulating MDSCs were correlated with early cancer relapse and predicted the prognosis of individuals with hepatocellular carcinoma undergoing therapeutic resection [63]. Furthermore, high levels of MDSCs were correlated with cancer stage and poor prognosis in bladder cancer patients, indicating that levels of MDSCs may serve as a prognostic biomarker in bladder cancer patients [64]. In diffuse large B-cell lymphoma patients, the expansion of M-MDSCs may be used as a biomarker for a worse prognosis. It is worthwhile mentioning that the accumulation of M-MDSCs in those patients was mediated by IL-35 [65]. Therefore, targeting M-MDSCs might be a potential therapeutic approach in patients with diffuse large B-cell lymphoma.

MDSCs have been observed to accumulate in CRC patients and tumor-bearing mice. As a result, several studies have concentrated on the immunosuppressive function of MDSCs and their characterization. In metastatic CRC, it was found that high numbers of G-MDSC were associated with poor prognosis [60]. Moreover, levels of circulating M-MDSCs were significantly elevated in most advanced tumor stages of CRC patients [66]. Additionally, levels of circulating M-MDSCs were significantly elevated after surgery, indicating that M-MDSCs levels are related to disease burden and tumor recurrence [66]. Similarly, expansion of circulating G-MDSCs was found to be correlated with higher stage and histological grade of malignancy, indicating their role in cancer progression [48]. Importantly, high levels of MDSCs were inversely correlated with vaccination responsiveness against the tumor antigen mucin 1 (MUC1) in patients with advanced colonic adenomas [67]. A recent study observed that level of PMN-MDSCs was significantly increased in the peritoneal cavity, blood, and spleen of CRC-derived peritoneal dissemination mice model [68], suggesting that targeted therapy of PMN-MDSCs would provide new therapeutic values for CRC-derived peritoneal dissemination.

## Targeting MDSCs

**Figure 2.**
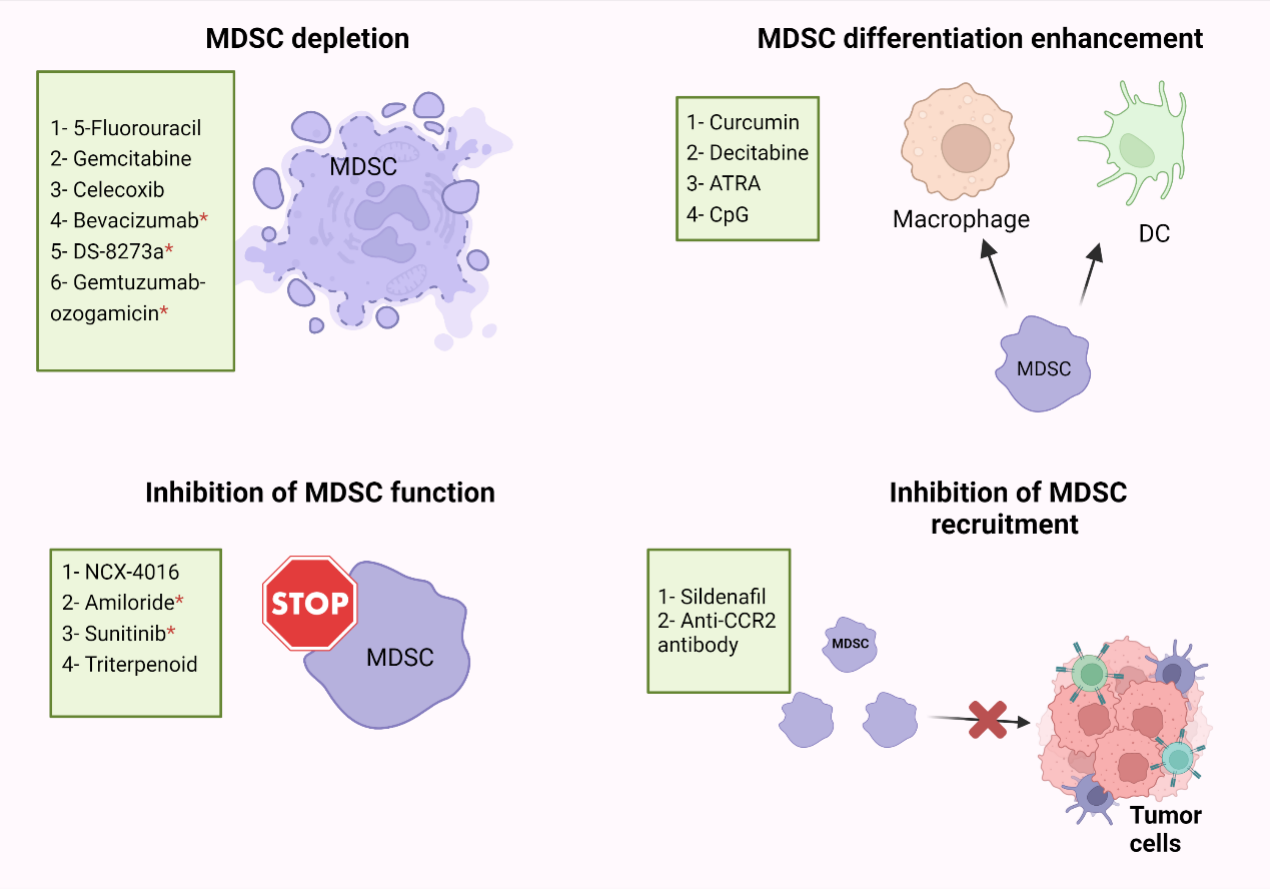
Potential strategies of MDSC targeting in human and mice models. MDSC-targeting agents are now being investigated in four major ways: MDSC depletion; inhibiting MDSC function; inhibiting MDSC recruitment; enhancing MDSC differentiation. ATRA: all-trans retinoic acid; CpG: cytosine-phosphate-guanosine; CCR2: C-C motif chemokine receptor 2; * human

Immune suppression plays important roles in the progression of tumors, and it is associated with the failure of cancer immunotherapies. MDSC levels were found to be positively correlated with cancer growth and progression, and their presence commonly reduces the efficiency of immunotherapies [69]. Some of the immune suppressive factors in the TME must be eliminated for cancer immunotherapy to be more effective [6, 69, 70]. Recently, several studies have described the use of different small molecules, nucleotides, conjugates, vitamins, and immunotherapeutic modalities, which are able to inhibit the suppressive activity of MDSCs in cancers [42, 71, 72], and achieve superior immunotherapeutic tumor control by radiotherapy [73]. However, there are limited studies on targeting MDSC in CRC patients, and we briefly discuss these studies below. Targeting of MDSC could be exploited in different ways including MDSC depletion, inhibiting MDSCs function and recruitment, or enhancing MDSCs differentiation, as described below and in Figure 2.

### MDSC depletion

Several studies have found that some cytotoxic anti-cancer agents could stimulate the immune system by depletion of MDSCs (Figure 2) [74–76]. 5-Fluorouracil (5-FU) and Gemcitabine have been found to specifically decrease the frequency of MDSCs in the spleen of animals bearing large tumors with no effect on T cells, DCs, natural killer (NK) cells, or B cells [75, 76]. Moreover, FOLFOX (folinic acid, 5-FU, and oxaliplatin) treatment protocol might be linked to a reduction in MDSC numbers and restoration of anti-tumor immunity, which resulted in less immunosuppression and a better clinical outcome in CRC patients [38, 77]. In addition, zoledronic acid suppressed tumor growth rate by inhibiting intratumoral MDSC accumulation in pancreatic cancer [78]. Also, Veltman et al. [79], found that treatment of mice carrying mesothelioma tumor cells with dietary celecoxib inhibited the local and systemic expansion of all MDSC subtypes.

Regarding CRC, it was reported that administration of celecoxib, a selective COX-2 inhibitor, significantly decreased the frequency of Gr1^+^CD11b^+^ immature myeloid-suppressor cells (I-MSCs) during chemoprevention of 1,2-dimethylhydrazine diHCl-(1,2-DMH) in CRC mice [80]. Moreover, treatment with gemcitabine reduced the tumor-suppressive environment by removing myeloid-derived CD11b^+^/Gr-1^+^ suppressor cells in breast cancer and CRC [81]. A recent study found that 5-FU suppressed sensitive MDSCs in CRC mouse models [82]. According to their hypothesis, some of the heterogeneous MDSCs are resistant to 5-FU, and 5-FU treatment removes only the sensitive MDSCs [82]. Moreover, administration of anti-VEGF (bevacizumab) was related with a reduction in the concentration of immature progenitor cells and generated a moderate rise in DC population in the peripheral blood of CRC patients [83]. Interestingly, high levels of MDSCs in the peripheral blood of CRC patients were reduced by using tumor necrosis factor-related apoptosis-inducing ligand receptor 2 (TRAIL-R2) agonistic antibody (DS-8273a) without affecting the frequencies of myeloid and lymphoid cell populations [84]. However, in numerous CRC patients, MDSC returned to pre-treatment levels by day 42 [84]. A recent study found that the anti-CD33 immunotoxin (gemtuzumab ozogamicin) could decrease MDSCs, opening the door for using it to reactivate T cells and chimeric-antigen receptor T cell (CAR-T) against various malignancies such as CRC, breast cancer, and melanoma [85].

### Inhibition of MDSC function and recruitment

MDSCs suppress the immune system by mainly producing two enzymes [ARG and nitric oxide synthase (NOS)], which metabolize the L-arginine. Nitric oxide (NO)-releasing aspirin (NCX-4016) was able to regulate the immunological state of hosts that had tumors, and it also increased the quantity and function of T cells that were specific for tumor antigens by suppressing ARG and NOS activities in the CRC mice model [86]. Moreover, amiloride, an antihypertensive drug, inhibited the formation of tumor-derived exosomes (TDEs) and reduced the suppressive functions of MDSCs in human CRC and mice models [87]. A study found that administration of sunitinib, a tyrosine kinase inhibitor, to several types of cancer including CRC, reduced phosphorylated STAT3 and ARG levels in M-MDSC, and increased T-cell proliferation [88]. Furthermore, treatment with synthetic triterpenoid C-28 methyl ester of 2-cyano-3,12-dioxooleana-1,9-dien-28-oic acid (CDDO-Me, bardoxolone methyl) was found to suppress the activity of MDSCs in a murine MC38 tumor host [89]. However, the proportions of MDSCs in the spleens were unaffected by this triterpenoid but their suppressive action was eliminated [89]. Another study demonstrated that sildenafil, a phosphodiesterase-5 inhibitor, regulated inflammation in the TME through inhibiting MDSC infiltration into tumor tissues in azoxymethane (AOM)/dextran sodium sulfate (DSS)-induced colonic tumorigenesis model [90]. Moreover, Liang et al. [91], found that treatment with anti-CCR2 antibody could decrease radiation-induced infiltration of M-MDSC in irradiated MC38 colon tumors.

### Enhancing MDSC differentiation

By knowing that the immune suppressive characteristics of MDSCs relate to their immature phase, pushing them to continue differentiation into mature myeloid cells could reduce their immune suppressive role (Figure 2) [92]. IL-12 [93], vitamin D3 [94], vitamin A [92, 95, 96], curcumin [97, 98], and cucurbitacin B [a selective inhibitor of Janus kinase 2 (JAK2)/STAT3] [99] are molecules that may induce MDSCs to differentiate into mature myeloid cells in several types of cancer [99]. In CRC mouse model, Daurkin et al. [100], demonstrated that tumor-infiltrating CD11b myeloid cells could be differentiated into mature myeloid cells in the presence of DNA demethylating agent 5-aza-2’- deoxycytidine (decitabine) (Figure 2). A recent study found that ATRA had potent activity in eliminating MDSCs by specifically up-regulating gene expression and protein level of glutathione synthase (GSS) in MDSCs, leading to their differentiation into mature myeloid cells in tumor-bearing mice (Figure 2) [96]. Moreover, another study showed that activation of Toll-like receptor-9 (TLR-9) by CpG (TLR-9 ligand) enhanced the maturation and differentiation of MDSCs, and effectively decreased the percentage of Ly6G^hi^ MDSCs in melanoma and CRC tumor models [101].

## Conclusions

In recent years, there has been increasing evidence that MDSCs play critical roles in modulating immune responses in a variety of pathological diseases, including cancers. A growing number of studies have reported the relationship between levels of MDSCs and tumorigenesis in CRC. MDSCs contribute to the advancement of CRC by suppressing anti-tumor activity. Obviously, levels of MDSCs correlate with the higher stage and histological grade of CRC. Based on available studies, we may need to categorize patients according to MDSC levels in peripheral and tumor tissues in order to select the most effective therapy and create innovative therapeutics for targeting MDSCs. Furthermore, monotherapies targeting MDSCs have shown some promise with limited success. Identifying these cells in human cancers is a challenge because of their wide heterogeneity. Further studies are needed to improve our understanding of MDSCs and their effects.

The origins of MDSCs, as well as the mechanisms by which they mediate immunosuppression, have recently been clarified. Obviously, several studies found that expansion of MDSCs in the periphery and tumor tissues was associated with poor prognosis. Unfortunately, the variety of cut-off values defining high levels of MDSCs in each study might be one of the studies’ limitations. In future studies, significant efforts should be made to explore if targeting certain MDSC subsets could have the potential to improve clinical outcomes in cancer patients.

## Abbreviations

5-FU: 5-Fluorouracil
ARG: arginase
CAC: colitis-associated cancer
CCL2: C-C motif chemokine ligand 2
CRC: colorectal cancer
CXCR2: C-X-C motif chemokine receptor 2
DCs: dendritic cells
e-MDSCs: early-stage myeloid-derived suppressor cells
GMCs: granulocytic myeloid cells
G-MDSCs: granulocytic myeloid-derived suppressor cells
IL-3: interleukin-3
MDSCs: myeloid-derived suppressor cells
M-MDSCs: monocytic myeloid-derived suppressor cells
PGE2: prostaglandin E2
PMN-MDSCs: polymorphonuclear myeloid-derived suppressor cells
PTEN: phosphatase and tensin homologue
RIPK3: receptor-interacting protein kinase 3
STAT3: signal transducer and activator of transcription-3
TME: tumor microenvironment
VEGF: vascular endothelial growth factor
YAP1: Yes-associated protein 1

## Declarations

### Copyright

© The Author(s) 2022.
